# Solid Lipid Nanoparticles for Dibucaine Sustained Release

**DOI:** 10.3390/pharmaceutics10040231

**Published:** 2018-11-14

**Authors:** Raquel de M. Barbosa, Ligia N. M. Ribeiro, Bruna R. Casadei, Camila M. G. da Silva, Viviane A. Queiróz, Nelson Duran, Daniele R. de Araújo, Patrícia Severino, Eneida de Paula

**Affiliations:** 1 Biochemistry and Tissue Biology Department, Institute of Biology, University of Campinas, Campinas 13083-862, SP, Brazil; nuneslica@gmail.com (L.N.M.R.); brucasadei@yahoo.com.br (B.R.C.); camilafarmacia1@yahoo.com.br (C.M.G.d.S.); vivianiqueiroz@yahoo.com.br (V.A.Q.); depaula@unicamp.br (E.d.P.); 2 Pharmacy Department, UNINASSAU—Natal College, Natal 59080-400, RN, Brazil; 3 Institute of Chemistry, University of Campinas (UNICAMP), Campinas 13083-861, SP, Brazil; nelsonduran1942@gmail.com; 4 Human and Natural Sciences Center, Federal University of ABC, Santo André 09210-580, SP, Brazil; daniele.araujo@ufabc.edu.br; 5 Institute of Technology and Research. Av. Murilo Dantas, 300, Aracaju 49032-490, SE, Brazil; pattypharma@gmail.com

**Keywords:** dibucaine, local anesthetics, solid lipid nanoparticles, drug delivery

## Abstract

Dibucaine (DBC) is among the more potent long-acting local anesthetics (LA), and it is also one of the most toxic. Over the last decades, solid lipid nanoparticles (SLN) have been developed as promising carriers for drug delivery. In this study, SLN formulations were prepared with the aim of prolonging DBC release and reducing its toxicity. To this end, SLN composed of two different lipid matrices and prepared by two different hot-emulsion techniques (high-pressure procedure and sonication) were compared. The colloidal stability of the SLN formulations was tracked in terms of particle size (nm), polydispersity index (PDI), and zeta potential (mV) for 240 days at 4 °C; the DBC encapsulation efficiency was determined by the ultrafiltration/centrifugation method. The formulations were characterized by differential scanning calorimetry (DSC), electron paramagnetic resonance (EPR), and release kinetic experiments. Finally, the in vitro cytotoxicity against 3T3 fibroblast and HaCaT cells was determined, and the in vivo analgesic action was assessed using the *tail flick* test in rats. Both of the homogenization procedures were found suitable to produce particles in the 200 nm range, with good shelf stability (240 days) and high DBC encapsulation efficiency (~72–89%). DSC results disclosed structural information on the nanoparticles, such as the lower crystallinity of the lipid core vs. the bulk lipid. EPR measurements provided evidence of DBC partitioning in both SLNs. In vitro (cytotoxicity) and in vivo (*tail flick*) experiments revealed that the encapsulation of DBC into nanoparticles reduces its intrinsic cytotoxicity and prolongs the anesthetic effect, respectively. These results show that the SLNs produced are safe and have great potential to extend the applications of dibucaine by enhancing its bioavailability.

## 1. Introduction

Effective pain management is one of the most difficult challenges in medicine. Local anesthetics (LA) are broadly used in algiatry to reversibly block neural transmission, curbing the pain sensation [1]. Chemically, LAs are amphiphiles whose potency and toxicity are directly related to the hydrophobic character of the compound [2].

In clinical practice, dibucaine (DBC), from the amine/amide family, differs from other commonly used LAs due to its large quinoline ring to which a butyl ether group is attached [3]. This rigid ring modulates DBC’s interaction with membranes, restricting the insertion of DBC to in-between the lipids [4]. In addition, DBC exhibits long-lasting action [5] and it is considered quite toxic to the central nervous and cardiac systems, with reports of convulsions, hypoxia, acidosis, arrhythmia, and cardiac arrest [6].

Solid lipid nanoparticles (SLN) are nanocarriers composed of an ordered solid lipid matrix encased by surfactant [7]. SLNs exhibit desirable physicochemical properties, biocompatibility, and the ability to encapsulate hydrophobic molecules [8,9]. The optimized pharmacokinetic and biological properties of LA-loaded SLNs have been reported for lidocaine, prilocaine, bupivacaine, and articaine [7,10,11,12]. In light of this, we considered encapsulating DBC in SLNs to enhance its bioavailability and to minimize its systemic toxicity [13,14].

DBC is commercially available in creams and ointments for cutaneous use. However, its use should be with caution due to its high toxicity; therefore, its use is limited [15]. These limitations have prompted the search for novel, sustained-release formulations to extend the DBC application range. This objective has led to the investigation of release systems for DBC that are capable of improving their availability [16].

In this work, dibucaine-containing SLN formulations prepared by two hot-emulsion preparation methods (high-pressure homogenization and sonication) were evaluated to ensure the reproducibility of SLN production. In addition, their cytotoxicity and the in vivo analgesic activity were tested. The formulations were aimed at providing a new delivery system for dibucaine, to be applied either topically or via infiltrative routes.

## 2. Materials and Methods

### 2.1. Materials

Dibucaine, poloxamer 188 (F68), and 5-doxyl-stearic acid spin labels (5-SASL) were obtained from Sigma-Aldrich (St Louis, MO, USA). Myristyl myristate (MM) and cetyl palmitate (CP) were from Dhaymers Fine Chemicals (São Paulo, Brazil) and Croda (São Paulo, Brazil), respectively. Other reagents used were HPLC grade: acetonitrile (J.T. Baker, Goias, Brazil), triethylamine (Vetec Rio de Janeiro, Brazil), and orthophosphoric acid (Cetus Ind. Com. Prod. Quim., Santo Amaro, Brazil). Deionized water (18.2 mΩ cm) was obtained from a Waters ultrapure water system (Merck KGaA, Darmstadt, Germany).

### 2.2. Methods

#### 2.2.1. Solid Lipid Nanoparticles: Preparation Methods

The hot-process emulsion method was used to prepare the SLN formulations. The lipids were weighed out in sufficient amounts to obtain a final concentration of 20 mg·mL^−1^. Myristyl myristate and cetyl palmitate were heated to 10 °C above their fusion points (38.0–41.6 °C and 43.0–54.0 °C for MM and CP, respectively) [7]. The anesthetic-loaded formulations were obtained by solubilizing DBC into these oily phases after their complete fusion up to a final 1 mg·mL^−1^ concentration, corresponding to 1:16 DBC:MM and 1:13 DBC:CP molar ratios, respectively. The oily phase was then slowly (over 3 min) added to a warm aqueous solution of poloxamer 188 (0.5%, *w*/*w*) under 10,000 rpm with a Turrax blender (IKA Werke, Staufen, Germany).

The obtained samples were then homogenized either using three cycles at 600 bar of a high-pressure hot homogenization process (H-P) in a Panda homogenizer (Niro Soavi, Parma, Italy) or by tip ultrasonication (U-S) at 20 kHz for 15 min in a Vibra-Cell ultrasonic processor (Sonics and Materials, Newtown, CT, USA). During the process, the sample media were insulated in order to keep the temperature above the melting point of the lipids. Finally, the SLNs were chilled to 20 °C and conditioned in glass flasks at 4 °C [17].

#### 2.2.2. Dibucaine (DBC) Quantification 

DBC concentration was measured using a Varian ProStar HPLC apparatus (Varian, Inc., Palo Alto, CA, USA) fitted with an OS 325 UV-vis detector, a PS 210 solvent delivery module, and an automatic injector. A reverse-phase C18 column (LiChroCART 100 RP-18, Merck KGaA, Hessen, Germany) was used with an acetonitrile:triethylamine phosphate buffer (55:45, *v*:*v*) mixture as the mobile phase, flow rate = 1.0 mL·min^-1^; DBC absorbance was detected at 247 nm [7,9].

#### 2.2.3. Encapsulation Efficiency Test

The encapsulation efficiency (%EE) of the SLN formulations for dibucaine was determined by the ultrafiltration/centrifugation technique, as previously described [9]. Each sample was diluted in Milli-Q water, added to an ultrafiltration unit (10 kDa cellulose, Millex, Millipore, Merck KGaA, Darmstadt, Germany), and centrifuged at 4000× *g* for 20 min (*n* = 3). The fraction of unencapsulated (free) DBC was quantified by HPLC using a calibration curve in the range 1.5–30.0 g·mL^−1^ [18]. The %EE was calculated according to Equation (1):

(1) $$\%\mathrm{EE}=\frac{A}{B}\times 100$$

where A is the amount of DBC entrapped in the SLN, and B refers to the initial amount of DBC in the samples; A = B − (free DBC).

#### 2.2.4. Determination of Solid Lipid Nanoparticles (SLN) Particle Size, Polydispersity Index, and Zeta Potential

Photon correlation spectroscopy (PCS) was used to measure the average particle size (nm) and polydispersity index (PDI) of the SLNs produced by the H-P or U-S methods. The zeta potential values were assessed with a Zetasizer Nano ZS analyzer (Malvern Panalytical Ltd., Royston, UK) at 25 °C. The samples were diluted in Milli-Q water prior to the analyses (1:100, *v*/*v*). For the zeta potential measurements, the samples were diluted in 0.1 mM sodium chloride solution (1:100, *v*/*v*) to ensure the formation of a compact electrical double layer. The analyses (*n* = 3) were repeated during storage (240 days at 4 °C), and the data are reported as mean ± standard deviation.

#### 2.2.5. Nanoparticle Tracking Analysis (NTA)

The size distribution and concentration of the nanoparticles produced by H-P were determined with a NanoSight LM20 instrument (NanoSight, Malvern Panalytical Ltd., Royston, UK) and NTA 2.0 software (NanoSight, Malvern Panalytical Ltd., Royston, UK) equipped with a laser diode (λ = 635 nm) [19]. The samples (*n* = 3) were diluted in Milli-Q water (1:5000, *v*/*v*), and the data are expressed as mean ± standard deviation. The particle size distribution, given as Span, was calculated using Equation (2):

(2) $$\mathrm{Span}=\frac{\left(D_{0.9}-D_{0.1}\right)}{D_{0.5}}$$

where D_0.9_, D_0.5_, and D_0.1_ are the width distribution based on 90, 50, and 10% of the cumulative particle size distribution frequencies of the nanoparticles, as assessed by NTA. 

#### 2.2.6. Transmission Electron Microscopy (TEM)

The morphologies of the SLN_M_ and SLN_CP_ particles (with and without DBC) produced by H-P were examined using a transmission electron microscope (Zeiss LEO-906, 60 kV, Carl Zeiss Microscopy, LLC, Thornwood, NY, USA). A small sample aliquot was added to a 200-mesh copper grid (Electron Microscopy Sciences, Hatfield, PA, USA) and left for 2 min. A drop of 2% (*w*/*w*) uranyl acetate solution was added onto the grid containing the sample to provide the contrast. Then, filter paper was used to remove all excess volume, and the samples were dried at room temperature.

#### 2.2.7. Differential Scanning Calorimetry (DSC)

Calorimetric analyses were performed on the SLNs prepared by the H-P method. The experiments were carried out using a DSC-Q10 system (TA instruments, New Castle, DE, USA); SLN samples were placed in aluminum sample holders and heated at a rate of 10 °C/min under a flow of N_2_ (50 mL·min^−1^). The crystallinity index (% CI) was calculated from the enthalpy (ΔH) of the thermal transition, taking the bulk lipid (MM, CP) as a reference (100% crystallinity), and according to Equation (3) [20]:

(3) $$\mathrm{CI}\%=\frac{\Delta H\;{\left(J/g\right)}_{\mathrm{nanoparticle}\left(\mathrm{SLN}\right)}}{\Delta H\;{\left(J/g\right)}_{\mathrm{lipid}\;\mathrm{bulk}\;\mathrm{enthalpy}\;\left(\mathrm{MM}\;\mathrm{or}\;\mathrm{CP}\right)}.\;\mathrm{lipid}\;\mathrm{phase}\;\mathrm{concentration}\;}\times 100$$

#### 2.2.8. Electron Paramagnetic Resonance (EPR)

The lipid milieu of the SLN formulations was analyzed from the spectra of the 5-SASL probe incorporated into the nanoparticles (with and without DBC), up to 1 mol % of the total lipid concentration. Spin labels were incubated with the sample for approximately 30 min. at 37 °C, and the EPR spectra were recorded using a Bruker EMX spectrometer (Bruker BioSpin GmbH, Billerica, MA, USA) at 20 °C. The interpretation of the EPR spectra considered the existence of lamellar structures inside the SLNs [21], in accordance with [22,23], in which the spin-label long molecular axis is roughly parallel to the bilayer normal. The order parameter (S), which provides information on the orientation of the probe molecule inserted in the lipid core, was calculated from the 5-SASL spectra according to Equation (4) [24]:

(4) $$S=\frac{2A//-2A⊥}{2\;\left[\mathrm{Azz}-\left(\mathrm{Axx}+\mathrm{Ayy}\right)/2\right]}$$

where A// and A⊥, directly measured in the EPR spectrum, are the hyperfine splitting for the spin label’s long molecular axis oriented parallel and perpendicular, respectively, to the external magnetic field. Azz (32 Gauss), Axx (6 Gauss), and Ayy (6 Gauss) were the principal components of the hyperfine tensor, measured in a single crystal. S values ranged from 0 to 1, with the unit revealing perfect anisotropy (parallel orientation to the bilayer normal) [25].

#### 2.2.9. In Vitro Release Experiments

The release of DBC (20 μg·mL^−1^) from the SLN formulations prepared by the H-P method was analyzed over time (48 h) at room temperature (25 °C), using 0.04 mol·L^−1^ phosphate buffer pH 7.4 in the acceptor compartment to guarantee the sink condition. At predetermined time intervals, the samples were filtered through cellulose membranes (Microcon, 10 kDa molecular exclusion size, Merck Millipore, Billerica, MA, USA) and centrifuged at 4100× *g* (MC 12V Sorvall centrifuge) for 20 min, prior to DBC quantification by HPLC.

#### 2.2.10. Mathematical Modeling of the Release Kinetic Curves

The kinetic curves can reveal significant information about the prevalent mechanisms ruling the release of drugs from drug-delivery systems. Among many tested mathematical models, the empirical Weibull and the semi-empirical Korsmeyer–Peppas [7] models were those that better fit the kinetic release curves of dibucaine from SLN samples. Equation (5) shows the Weibull model, adapted from [26], that considers the cumulative fraction of released drug as a function of time (*t*) [27].

(5) log[−ln(1−m)]=βlog(t−Ti)−logα

where *α* is the time interval prior to the beginning of the release process; Ti is the initial release time; and *β* is the shape parameter of the exponential curve. *β* > 1 describes a sigmoid (fast kinetics) curve; *β* = 1 is related to first-order kinetics; *β* < 1 indicates satellite (slow kinetics); and *β* < 0.75 denotes Fickian diffusion. 

Korsmeyer and Peppas [28] proposed Equation (6), which exponentially relates drug release levels and time. The release exponent (*n* value) explains the mechanism of drug release as a function of time, *t*. Depending on the n value, it is possible to infer the prevalent release mechanism from drug-delivery systems: *n* < 0.43 is related to Fickian diffusion; *n* > 0.85 describes type II transport (from swellable and relaxable matrixes); and 0.43 < *n* < 0.85 is found in the case of anomalous transport kinetics [29].

(6) $$f_{t}=at^{n}$$

where *n* = release exponent, $f_{t}$ = the amount of drug released at time *t*.

#### 2.2.11. In Vitro Cytotoxicity

Cytotoxicity tests were performed using mouse embryo BALB/c 3T3 fibroblasts (National Institute of Health, Baltimore, MD, USA) and immortalized human keratinocytes, HaCat cells (Academic Medical Center, Amsterdam University, Amsterdam, The Netherlands). Cell viability was assessed by the reduction of 3-(4,5-dimethylthiazol-2-yl)-2,5-diphenyltetrazolium bromide (MTT) to formazan (MTT test) [30]. Samples containing different concentrations of DBC (0.02–4.20 mmol·L^−1^), free or encapsulated in the SLNs, were added to the culture medium. After 3 h of incubation, the treatment medium was removed and replaced by a solution (0.5 mg·mL^−1^) of MTT. After a 2 h incubation at 37 °C, the medium was removed, and ethanol (100 μL) was added to each well in order to solubilize the formazan. After 10 min of agitation, the samples were quantified at 570 nm in a BIO-TEK Elx 800 spectrophotometer (BioTek Instruments, Inc., Winooski, VT, USA). The values are expressed as the MTT reduction percentage from *n* = 6 measurements [9].

#### 2.2.12. *Tail Flick* Test

The *tail flick* test described by D’Amour and Smith [31] was used to evaluate the antinociceptive activity of the samples topically applied to the tail base region of male adult Wistar rats (*Rattus norvegicus albinus*). The animals (250–300 g) were obtained from CEMIB (Centro de Bioterismo da Unicamp), which is certified by the International Council for Laboratory Animal Sciences (ICLAS). Wistar rats were subjected to light/dark cycles of 12 h, with water and food *ad libitum*, and monitored at room temperature (22 ± 3 °C) for 7 days. All the experiments followed the Ethical Principles in Animal Experimentation, adopted by the Brazilian Society of Laboratory Animal Science (SBCAL). The protocols were approved by the Ethics Committee on Animal Research (CEUA) of the Biology Institute of the UNICAMP (Protocol #2464-1, 04.07.2011).

Briefly, the animals were placed in an individual acrylic container (horizontal position) after being placed in a restraint over an analgesimeter with a portion of the tail (5 cm from its tip) exposed to heat from a projector lamp (55 ± 1 °C; 150 W); a 15 s cutoff time was used to avoid thermal injury, and the baseline (normal response to the noxious stimulus) was established. For analgesia of the caudal nerve, the formulations containing free or encapsulated DBC at 0.05% were topically applied to the back of the rat’s tail. The analysis started 30 min after the sample’s administration, and the response was recorded by a control switch and a timer, simultaneously activated. The timer was stopped immediately when the rat tail flicked. The results obtained were expressed as the percentage of maximum possible effect (MPE%) according to Equation (7) [32]:

(7) $$\mathrm{MPE}\%=\frac{\mathrm{latence}\;\mathrm{time}-\;\mathrm{basal}\;\mathrm{line}\;}{\mathrm{cut}\;\mathrm{off}-\mathrm{basal}\;\mathrm{line}}\times 100$$

The same observer performed all experiments. The recovery time from anesthesia induced by either free DBC or SLN formulations was calculated according to Equation (8):

(8) $$\Delta T_{\mathrm{rec}}=\frac{T_{\mathrm{REC}\;\mathrm{encapsulated}\;\mathrm{DBC}}-T_{\mathrm{REC}\;\mathrm{free}\;\mathrm{DBC}}}{T_{\mathrm{RECfree}\;\mathrm{DBC}}}\times 100$$

where $T_{\mathrm{REC}\;\mathrm{encapsulated}\;\mathrm{DBC}}$ is the recovery time of the animals after application of the DBC-containing SLN formulations; $T_{\mathrm{REC}\;\mathrm{free}\;\mathrm{DBC}}$ is the recovery time after application of free dibucaine.

#### 2.2.13. Statistical Analyses

Statistical data analyses were performed by Student’s *t*-test, ANOVA, and Tukey post hoc tests with a significance level of 5% (*p* < 0.05). The data were calculated using Instat v. 3.0 software (GraphPad, San Diego, CA, USA, 1997).

## 3. Results

### 3.1. Characterization of SLNs

SLN formulations were prepared with MM or CP as solid lipids plus Pluronic F68 as surfactant and using one of the two different homogenization techniques: H-P or U-S. The nanoparticle’s size (nm), polydispersity index (PDI), and zeta potential (mV) are shown in Table 1. In general, the size of SLNs ranged from 188 to 288 nm, with or without DBC. For all the samples tested, the PDI values were low (0.14–0.27), while the zeta values showed larger fluctuations, from −3 to −45 mV. The statistical analyses (using ANOVA and Tukey’s test) revealed no significant differences between the samples containing or not containing DBC (*p* > 0.05), except for SLN_CP_/U-S vs. SLN_CP_DBC/U-S, in which the zeta potential decreased in modulus after DBC incorporation (Table 1).

H-P and U-S methods produced quite similar SLNs. The average diameter of the nanoparticles produced by H-P was always slightly smaller than those prepared by U-S, with no statistically significant differences. For the SLN_CP_ formulations (with and without DBC), the PDI values were significantly smaller when H-P was used, and the zeta potentials were higher in modulus (SLN_CP_/H-P only).

As expected, all the tested formulations showed high DBC encapsulation efficiency, ranging from 72.3 to 89.3%. The highest encapsulation efficiency (~89.3%) was found with SLN_MM_DBC/U-S. Significant differences were observed between the SLN_CP_ samples prepared by H-P or U-S.

**Figure 1:**
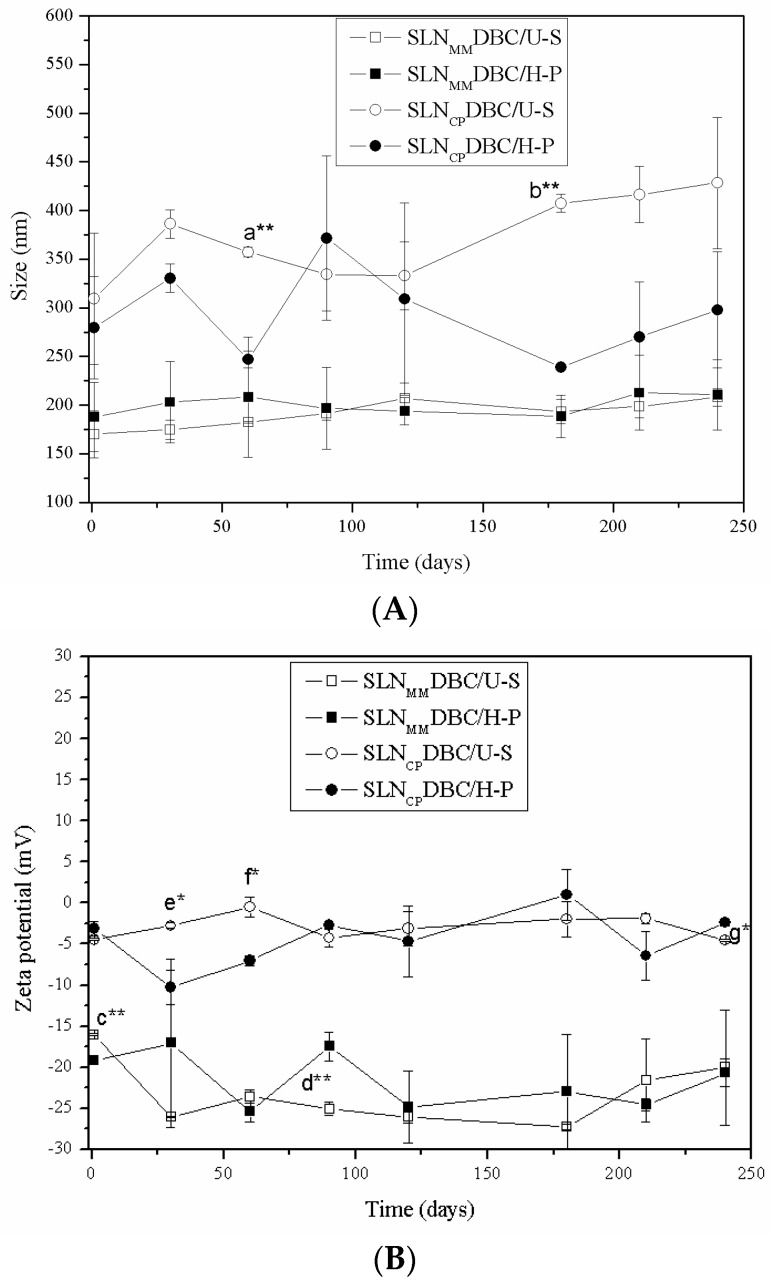
Stability study of SLNs containing DBC, prepared by high-pressure (H-P) or sonication (U-S) methods, during storage at 4 °C for 240 days. Stability is measured in terms of particle size (**A**) and zeta potential values (**B**). Statistical analysis: comparison between days 1 and 240 after preparation (ANOVA) and between the processes used to produce the SLN (Student’s *t*-test, *p* < 0.05). *p* < 0.001 (***); *p* < 0.01 (**); *p* < 0.05 (*); not significant (ns). Difference between days 1 and 240: a. SLN_CP_DBC/U-S vs. SLN_CP_DBC/H-P (60 days of storage); b. SLN_CP_DBC/U-S vs. SLN_CP_DBC/H-P (180 days of storage). Zeta potential: c. SLN_MM_DBC/U-S vs. SLN_MM_DBC/H-P (1 day of storage); d. SLN_MM_DBC/U-S vs. SLN_MM_DBC/H-P (90 days of storage); e. SLN_CP_DBC/U-S vs. SLN_CP_DBC/H-P (30 days of storage); f. SLN_CP_DBC/U-S vs. SLN_CP_DBC/H-P (60 days of storage); g. SLN_CP_DBC/U-S vs. SLN_CP_DBC/H-P (240 days of storage). Data are shown as mean ± standard deviation (*n* = 3).

All the systems remained stable for 240 days of storage at 4 °C regarding size, PDI, and zeta potential, except for SLN_CP_DBC (prepared by H-P or U-S), whose zeta values became significantly less negative (*p* < 0.05). The results in Figure 1 show the appropriateness of both (H-P and U-S) methodologies in the preparation of stable SLN formulations [33].

Encapsulation efficiency (%) was evaluated at 1 to 240 days after sample preparation. After 240 days elapsed, a decrease of 30 and 40% in the amount of encapsulated DBC over the storage time was observed for SLNCPDBC and SLNMMDBC, respectively.

Table 2 shows results obtained by NTA for the nanoparticles prepared by the H-P method. The polydispersity of the nanoparticles size is expressed by the Span value [19], as described in Methods.

As expected, the average SLN sizes determined by NTA were always smaller than those determined by PCS (Table 1) [34]. Moreover, no significant changes in the particles size were observed after DBC encapsulation, in agreement with the data in Table 1. The above-mentioned results directed the choice of SLN formulations prepared by the H-P method for use in subsequent sample preparations.

**Figure 2:**
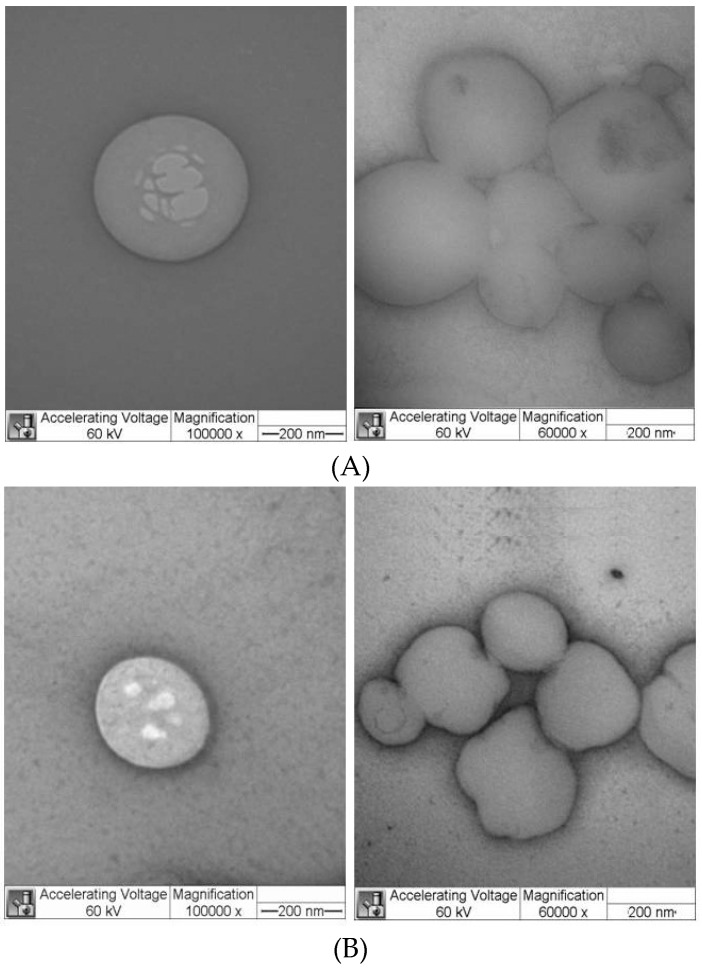
TEM micrographs of the SLN formulations SLN_CP_ (**A**) and SLN_CP_DBC (**B**), prepared by high-pressure homogenization, at two different magnifications: 100,000× (**left**) and 60,000× (**right**). CP: cetyl palmitate.

Figure 2 shows representative TEM micrographs (of SLN_CP_, with and without DBC) prepared by H-P homogenization. Similar images were obtained with SLN_MM_ and SLN_MM_DBC. The nanoparticles showed a spherical morphology and delimited contours, with particles sizes around 200–250 nm. Additionally, the incorporation of DBC did not change the morphology and size of the nanoparticles, which is in good agreement with the PCS and NTA data.

**Figure 3:**
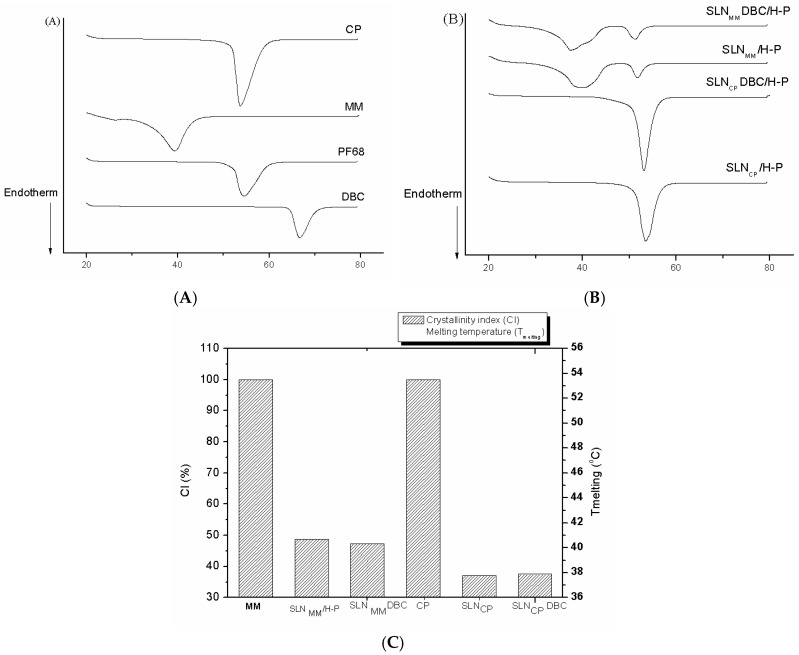
Differential scanning calorimetry (DSC) analyses of SLN formulations prepared by high-pressure homogenization: (**A**,**B**) show thermograms of SLN samples and their excipients, respectively. (**C**) Crystallinity index of the formulations, as calculated according to Equation (3).

The structural characterization of the SLN formulations was first evaluated by DSC. Figure 3 shows the thermograms of excipients and SLN formulations prepared by high-pressure homogenization, as well as the calculated crystallinity index (see Methods). The corresponding melting points and enthalpy values for the data in Figure 3A are provided in Table S1 (Supplementary Material). DBC has an endothermic peak at 66.7 °C, corresponding to its melting point [35], which was not observed in the thermogram of DBC-loaded SLNs (Figure 3A). The melting points of the excipients (MM, CP, and Pluronic F68) are clearly seen at 39.3, 53.7, and 54.6 °C, respectively. These peaks can also be observed in the corresponding (SLN_MM_ and SLN_CP_) formulations, and the addition of DBC did not appear to affect such excipient transitions in any of the formulations (Figure 3B).

The thermal behavior of pure MM and CP were also used to assess the crystallinity of these excipients and SLN matrix [20,36]. Figure 3C shows the crystallinity index (CI), which was assessed for all the SLN formulations after 10 days of preparation and calculated according to Equation (3). The high CI values of MM and CP (Figure 3C) reflect the crystalline arrangement of these lipids, while the lower CI of the SLN_MM_ and SLN_CP_ formulations (with and without DBC) reveals the substantial rearrangement of the bulk lipids inside the nanoparticles (CI < 50%) toward a less organized arrangement. 

**Figure 4:**
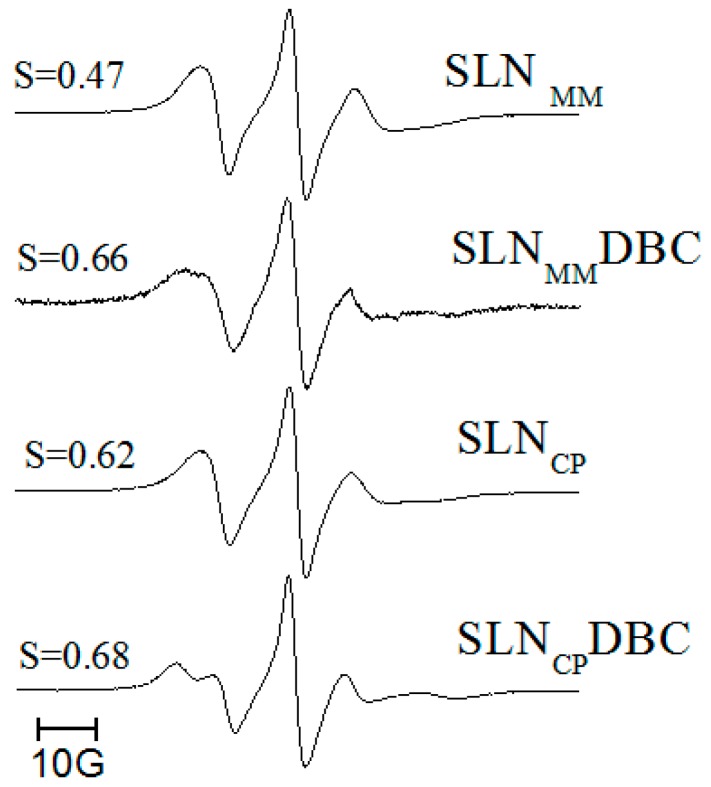
Structural characterization of SLN formulations prepared by high-pressure homogenization. Electron paramagnetic resonance (EPR) spectra of 5-SASL inserted into SLN_MM_, SLN_MM_DBC, SLN_CP_, SLN_CP_DBC, measured at 20 °C. The segmental order parameter, S, is also given (see text). MM: myristyl myristate.

To assess the effect of DBC on the mobility and organization of the lipid core of the nanoparticles, we conducted EPR measurements. The 5-SASL probe was incorporated into the lipid milieu of the SLNs to monitor the molecular arrangements of MM and CP. When inside bilayers, 5-SASL monitors regions closer to the polar head group due to the high polarity of the stearic acid, and since the nitroxide paramagnetic probe is linked to the fifth carbon of its acyl chain [37]. Also, in the bilayers, the segmental order parameter (S), calculated from 5-SASL and describing the orientation of the spin probe molecule, ranges from 0 (in disordered, isotropic systems) to 1 (in anisotropic, completely oriented systems) [25]. Figure 4 shows the spectra of 5-SASL incorporated into the SLN formulations, in the presence or absence of DBC, measured at 20 °C. The spectra are compatible with the existence of lamellar lipid arrangements, allowing for the determination of the segmental order parameter (S). S values increase in the presence of dibucaine, showing that the anesthetic disturbs (increases the anisotropy of) the lipid milieu monitored by the spin label probe.

### 3.2. In Vitro Release Profile of DBC

**Figure 5:**
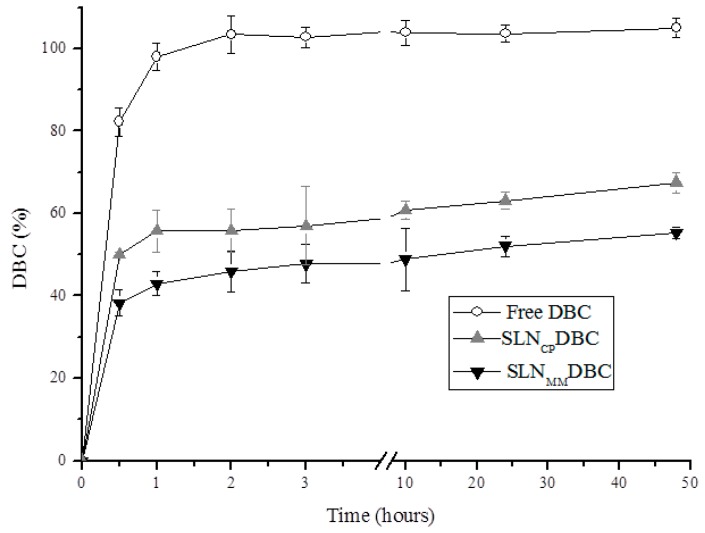
Kinetic curves for the in vitro release of DBC, free or encapsulated in SLNs, prepared by H-P homogenization (mean ± SD, *n* = 3) at room temperature (25 °C).

The release profile [38,39] of the DBC-loaded SLNs prepared by H-P is shown in Figure 5. An initial burst effect is observed, followed by the sustained release of DBC for over 48 h. On the other hand, free DBC (control) is completely released from the donor to the acceptor compartment within the first 2 h of the experiment (Figure 5).

In the present study, the values found for the release constant using the mathematical models proposed by Korsmeyer and Peppas (semi-empirical equation) and Weibull (empirical equation) (*β* ≤ 0.75 and *n* ≤ 0.43, respectively—see Equations (5) and (6) in Methods) revealed, in both cases, that Fickian diffusion was the prevalent release mechanism of dibucaine from the nanoparticles. 

### 3.3. In Vitro Cytotoxicity

**Figure 6:**
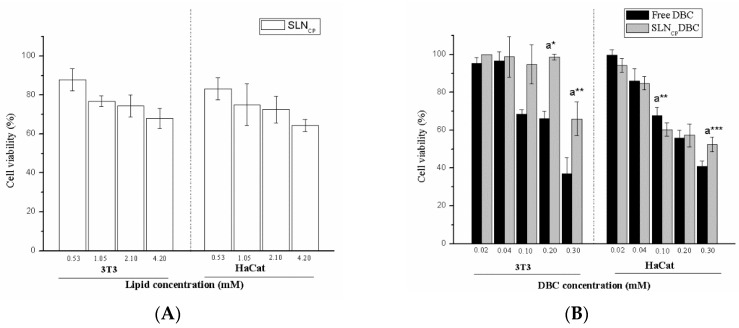
Cell viability tests using 3T3 fibroblasts and HaCaT keratinocytes. (**A**) Cells treated with SLN_CP_ prepared by high-pressure homogenization; (**B**) cells treated with DBC, free and encapsulated in the SLN_CP_ (mean ± SD, *n* = 6). a. Free DBC vs. SLN_CP_DBC, * *p* < 0.05, ** *p* < 0.01, *** *p* < 0.001 (Student’s *t*-test, 95% confidence level).

The cytotoxic effect of the SLN_CP_ formulations, with and without DBC, against 3T3 fibroblasts and HaCaT cells is given in Figure 6. Similar tests have been previously conducted for SLN_MM_ formulations [9]. Under the experimental conditions, the control SLNs (in the absence of DBC, Figure 6A) were noncytotoxic to the cells, even at high concentrations (up to 4.2 mmol·L^−1^). Free DBC was found to be cytotoxic to both cell lines, with IC_50_ values in the range of 0.2–0.3 mmol·L^−1^. Encapsulation of DBC into the SLN_CP_ formulation significantly decreased its cytotoxic effect against 3T3 cells (*p* < 0.05). In HaCaT cells, no significant changes were observed in low-concentration samples, but a tendency toward increased cell viability was detected (SLN_CP_DBC vs. free drug) mainly at high DBC concentrations (*p* < 0.0 and *p* < 0.001, at 0.1 and 0.3 mM, respectively) (Figure 6B). The lower sensitivity of HaCaT cells than fibroblast cells to xenobiotics has been previously reported [40], and it was confirmed here by the slightly higher IC_50_ values determined after treatment with free DBC than those determined for 3T3 cells (Figure 6B). 

### 3.4. Tail Flick Test

**Figure 7:**
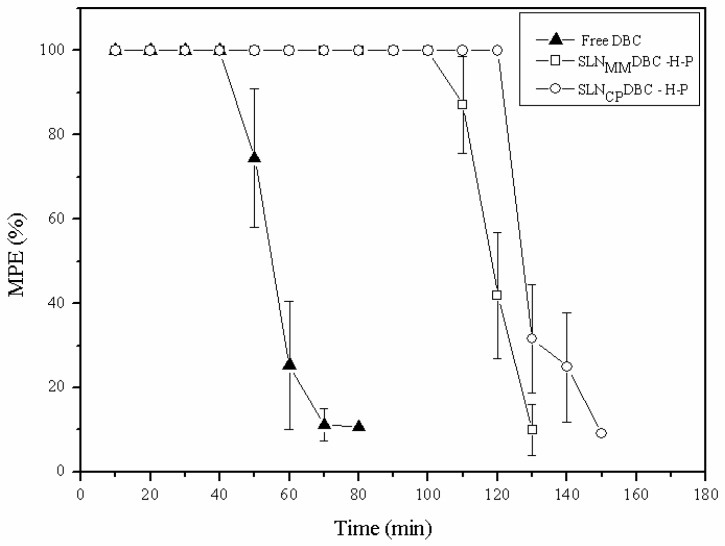
Maximum effect percent (analgesic activity) vs. time, assessed by the *tail flick* test (*n* = 7 animals per group) after administration of dibucaine, free or encapsulated in SLN_CP_/H-P and SLN_MM_/H-P.

The duration (recovery time) and maximum antinociceptive effect of the formulations are given in Table 3 and Figure 7, respectively. The formulations (SLN_MM_DBC/H-P and SLN_CP_DBC/H-P) doubled the recovery time (100.0% and 115.4%, respectively) compared to that induced by free DBC at the same (0.05%, *m*/*v*) concentration.

## 4. Discussion

SLN formulations composed of MM or CP as solid lipids, prepared by two different homogenization techniques (high-pressure and tip sonication), were tested in order to observe the influence of the composition and preparation method on the stability and DBC encapsulation efficiency. The solid lipids (MM, CP) were selected for their biocompatibility and spread use, and also because of their low fusion points, which enabled easier preparation of the particles at relatively mild temperatures without thermal degradation [7,9,41].

Testing different methodologies is justified when scale-up procedures are considered, since the structural properties of laboratory-prepared SLN should be kept in large-scale production. The particle size and PDI values found for the SLNs prepared by the H-P and U-S methodologies reveal a monodisperse distribution of nanoparticles of suitable sizes (ca. 200 nm) in both kinds of formulations (MM and CP) [42], attesting to the reproducibility of the methods.

Interestingly, the surface charges of the nanoparticles become less negative following DBC addition in all evaluated systems. Since DBC is mainly protonated at the pH of the formulations [43], the less negative zeta values may be taken as evidence of DBC insertion inside the SLN. Except for SLN_CP_DBC/U-S (for which the nanoparticle size increases over the time—Figure 1), the PCS results reveal the great stability of the systems after 240 days of storage at 4 °C, although a slight decrease in %EE over the storage time is observed for both SLNs. These results agree with reports from other researchers [44,45,46] that attribute the retention capacity of drugs in lipid nanoparticles to the production method and lipid composition. Particles with an imperfect crystalline arrangement (with lipid crystallization altered by small amounts of oil), an amorphous structure (solid but not crystalline matrix), or a multiple crystalline structure (with the solid lipid matrix containing oily nanocompartments) may favor the entrapment of the drug. In this work, no liquid lipids (at room temperature) were present in the lipid core of the nanoparticles, explaining the decrease in %EE over time.

Stable systems show the compatibility between the excipients and DBC [7], where Pluronic F68 serves as a steric stabilizer to avoid aggregation [10,11]. In the case of SLN_CP_DBC/U-S, instability suggests a redistribution of the encapsulated DBC during the storage period.

The PCS results reveal that the H-P and U-S methodologies are equally appropriate for the preparation of SLNs, and the encapsulation efficiency of the formulations prepared either by H-P or U-S is also equivalent. Therefore, considering that high-pressure homogenization is a more suitable method for scale-up compared to tip sonication [33], subsequent tests were conducted only with SLNs prepared by high-pressure homogenization.

Complex systems such as SLNs should be tracked over time by different analytical techniques to provide a robust data set [19]. To this end, the morphology and the hydrodynamic diameters of the SLNs produced by high-pressure homogenization were confirmed by TEM and nanotracking analysis. In this last technique, the Brownian motion of the nanoparticles was monitored individually by recording the scattered light. Dispersed and spatially resolved particles were visualized by video recording using a charge-coupled device camera. The largest particles appear as bright spots, and the smaller particles move more rapidly, as expected due to their Brownian motion [47,48]. Different intensities of light scattering are observed for each formulation, but the cumulative percentiles of the particle diameter distributions indicate the existence of single populations of particles. Unlike the PCS data, NTA values are not affected by the average vesicle diameter, and could, therefore, be used to validate the PCS data [19].

The DSC results provide information on the thermodynamic properties of SLNs. As expected, SLNs present an endothermic transition near the melting point of their major components (MM or CP) but with decreased peak enthalpies. The lower enthalpies in SLNs than in the bulk lipids could be explained by the nanoparticle size (increased specific surface area), interaction with compounds of the formulation, or the presence of other polymorphic forms. In addition, all DBC-loaded SLNs exhibit melting points (>50 °C) above body temperature, which is a prerequisite for retaining the solid state of the nanoparticles [49]. In fact, many of the claimed advantages of SLNs as drug-carrier systems are related to the solid state of its lipid matrix, with lipid crystallinity being a determinant for the performance of these carriers [50,51]. The broadening of the transition peaks, the decreased enthalpies, as well as the slightly lower melting points observed in SLN samples (in comparison to MM and CP) discloses the lower organization of the nanoparticles’ lipid core [36], which is confirmed by their significantly lower CI (Figure 3C). Such a decrease in crystallinity is desirable because it is associated with a reduced rate of loss of the active agent from the carrier during storage, increasing the shelf life of the product [50,51].

EPR provides more detailed information on the organization of the lipid core of the SLNs. The spectra of 5-SASL reveal the presence of lamellar lipid arrangements inside the nanoparticles, as also confirmed by small angle X-ray diffraction [21]. As expected, the order parameter of the SLN_CP_/H-P is higher than that of SLN_MM_/H-P, as expected from CP’s larger acyl chain (C16) in comparison to MM (C14). More interestingly, the S values reveal that when dibucaine is incorporated into both formulations (SLN_MM_DBC/H-P and SLN_CP_DBC/H-P), it favors the rearrangement of the lipids in the region monitored by the spin-label probe toward a more organized environment (higher S values), clearly indicating that the anesthetic molecule is incorporated into the lipid core of the SLNs.

The in vitro drug release studies provide useful information about the distribution of the encapsulated agent. The two DBC-loaded SLNs (Figure 5) show a biphasic release profile, i.e., an initial burst effect (~30 min) that is desirable for the onset of action of local anesthetics [12], followed by a sustained DBC release over the remaining 48 h of the experiment. The burst effect is due to the non-encapsulated DBC fraction in the SLN formulations [7], while Fickian diffusion governs the sustained release curves, according to the Weibull and Korsmeyer and Peppas models [48]. The DBC release profile from SLNs is in agreement with other reports in the literature [38,39] that suggest that drugs are solubilized throughout the solid lipid matrix of nanoparticles.

In vitro cytotoxicity tests were conducted in fibroblasts (3T3) and keratinocytes (HaCaT), which are useful cell models for the identification of substances that are skin irritants [52]. Both cell lineages are found to be sensitive to the effect of free DBC, and the toxicity drops (IC_50_ values were never reached) after the encapsulation of DBC in the SLN_CP_ formulation. These results are in agreement with our previous observations on the effect of DBC encapsulated in SLN_MM_ and nanostructured lipid carriers [9].

Once the safety of SLN formulations was confirmed for all the cell lines tested, the in vivo antinociceptive activity was assessed by the *tail flick* test. The SLN_MM_DBC/HP and SLN_CP_DBC/HP formulations increase (100% and 115%, respectively) the recovery time from anesthesia, in comparison to free DBC. This effect reflects the high encapsulation efficiency of the nanoparticles and agrees with the in vitro sustained release of dibucaine. As a result, the therapeutic action of the formulations is 2 times higher than that of free DBC.

## 5. Conclusions

Solid lipid nanoparticles containing dibucaine were prepared using myristyl myristate or cetyl palmitate as solid lipids and Pluronic F68 as the surfactant by two (H-P and U-S) hot-emulsion techniques. The formulations show good stability and high encapsulation efficiency (over 70%), and H-P formulations were characterized using a set of methods (DSC, EPR, TEM). Encapsulation into SLN enhances drug solubility, promotes the sustained release of DBC, reduces its intrinsic toxicity against 3T3 and HaCaT cells, and doubles the anesthetic’s effect in rats, in comparison to the control. Altogether, these achievements support the potential of SLN-based drug delivery systems to be used with dibucaine not only for topical anesthesia but perhaps by infiltration routes since, to date, there are no commercial products for infiltrative anesthesia using this anesthetic agent.

## Figures and Tables

**Table 1:** Structural properties of solid lipid nanoparticle (SLN) formulations prepared by high-pressure (H-P) or ultrasonic (U-S) homogenization. Photon correlation spectroscopy measurements on freshly prepared samples are shown in terms of size (nm), polydispersity index (PDI), and zeta potential values. Data are presented as mean ± standard deviations (*n* = 3).

Nanoparticle	Size (nm)	PDI	Zeta Potential (mV)	EE (%)
SLN_MM_/U-S	227.80 ± 3.80	0.22 ± 0.05	−25.13 ± 7.62	
SLN_MM_DBC/U-S	250.17 ± 61.40	0.21 ± 0.07	−19.18 ± 3.73	89.3 ± 3.5 ^g^***
SLN_MM_/H-P *	188.02 ± 7.07	0.15 ± 0.02	−26.91 ± 7.72	
SLN_MM_DBC/H-P *	234.33 ± 42.87	0.23 ± 0.06	−18.47 ± 2.55	76.6 ± 7.9
SLN_CP_/U-S	287.75 ± 15.20	0.32 ± 0.01 ^a^**	−26.03 ± 6.50 ^d^*	
SLN_CP_DBC/U-S	271.60 ± 30.72	0.27 ± 0.02 ^b^***	−10.67 ± 1.53	72.3 ± 4.2
SLN_CP_/H-P	239.37 ± 18.31	0.18 ± 0.03 ^c^***	−45.93 ± 4.64 ^e^***^,f^**	
SLN_CP_DBC/H-P	262.63 ± 48.42	0.14 ± 0.03	−3.89 ± 0.96	78.4 ± 5.0

* Results from reference [9]. Statistical analyses: ANOVA and Turkey–Kramer test, *p* < 0.001 (***), *p* < 0.01 (**), *p* < 0.05 (*). PDI: ^a^ SLN_CP_/U-S vs. SLN_CP_DBC/U-S; ^b^ SLN_CP_DBC/U-S vs. SLN_CP_DBC/H-P; ^c^ SLN_CP_/U-S vs. SLN_CP_/H-P. Zeta potential: ^d^ SLN_CP_/U-S vs. SLN_CP_DBC/U-S; ^e^ SLN_CP_/H-P vs. SLN_CP_DBC/HP; ^f^ SLN_CP_/U-S vs. SLN_CP_/H-P. EE%: ^g^ SLN_MM_DBC/H-P vs. SLN_MM_DBC/U-S. DBC: dibucaine; EE: encapsulation efficiency.

**Table 2:** Sizes (Z-averages) and polydispersity (Span) for the SLN formulations prepared using high-pressure (H-P), as determined by nanoparticle tracking analysis (NTA), 24 h after sample preparation.

Nanoparticle/Procedure	Cumulative Diameter Distribution (D)			Z-Average (nm)	Span
	D_10_ (nm)	D_50_ (nm)	D_90_ (nm)		
SLN_MM_/H-P	118	178	248	172.00 ± 7.81	0.7
SLN_MM_DBC/H-P	119	174	243	175.67 ± 10.69	0.7
SLN_CP_/H-P	96	181	255	173.33 ± 5.13	0.9
SLN_CP_DBC/H-P	80	148	234	178.00 ± 9.54	1.0

Data are presented as means ± standard deviations (*n* = 3). D_10_, D_50_, and D_90_ refer to the diameters based on 10, 50, and 90% of the cumulative distribution. Statistical analysis using the Student’s *t*-test: not significant at *p* > 0.05.

**Table 3:** Duration of the analgesic effect (recovery time) induced by dibucaine, free or encapsulated into the SLN formulations, measured by the *tail flick* test in Wistar rats. Data are presented as means ± standard deviations, *n* = 7.

Samples	*T_REC_* (min)	Δ*T_REC_* (%)
Free DBC	65.0 ± 12.9 ^a^***^,b^***	-
SLN_MM_DBC/H-P	130.0 ± 10.0	100.00
SLN_CP_DBC/H-P	140.0 ± 10.0	115.38

Statistical analyses: ^a^ Free DBC vs. SLN_MM_DBC/H-P, ^b^ Free DBC vs. SLN_CP_DBC/H-P *** *p* < 0.001 (Student’s *t*-test, 95% confidence level; ANOVA with Tukey–Kramer test).

## Supplementary Materials

The following are available online at http://www.mdpi.com/1999-4923/10/4/231/s1, Table S1: Melting points and enthalpies (∆H) obtained for the SLN formulations prepared by H-P, and their components, measured by differential scanning calorimetry.

## Abbreviations

5-SASL	doxyl-stearic acid spin labels
CI	crystallinity index
CP	cetyl palmitate
DBC	dibucaine
DSC	differential scanning calorimetry
EE	encapsulation efficiency
EPR	electron paramagnetic resonance
HaCat cells	immortalized human keratinocytes
H-P	high pressure
MM	myristyl myristate
MTT	3-(4,5-dimethylthiazol-2-yl)-2,5-diphenyltetrazolium bromide
NTA	Nanoparticle tracking analysis
PCS	photon correlation spectroscopy
PI	polydispersity index
Size	Z-average
SLN	solid lipid nanoparticles
TEM	transmission electron microscopy
U-S	ultrasonication
