# Supplementary material for: Solid Lipid Nanoparticles for Dibucaine Sustained Release
Source: Pharmaceutics. 2018 Nov 14;10(4):231. doi: 10.3390/pharmaceutics10040231 (PMC6321380; doi:10.3390/pharmaceutics10040231)
Supplement: Supplementary file 1 [file pharmaceutics-10-00231-s001.pdf]

pharmaceutics

Supplementary Materials: Solid Lipid Nanoparticles for Dibucaine Sustained Release

Raquel de M. Barbosa, Ligia N.M. Ribeiro, Bruna R. Casadei, Camila M. G. da Silva, Viviane A. Queiróz, Nelson Duran, Daniele R. de Araújo, Patrícia Severino and Eneida de Paula [1]

Table S1. Melting points and enthalpies (ΔH) obtained for the SLN formulations prepared by H-P, and their components, measured by differential scanning calorimetry.

Samples	Melting Point (°C)	ΔH (J/g)
DBC	66.7	−94.3
Pluronic F68	54.6	−145.6
Myristyl myristate	39.3	−179.5
Cetyl palmitate	53.7	−212.1
SLN_{MM}/H-P	39.8	−65.6
SLN_{MM}DBC/H-P	37.4	−69.3
SLN_{CP}/H-P	53.4	−66.7
SLN_{CP}DBC/H-P	53.1	−65.4
